# Dibenzyl­aza­nium (oxalato-κ^2^ *O*,*O*′)triphenyl­stannate(IV)

**DOI:** 10.1107/S1600536812021125

**Published:** 2012-05-31

**Authors:** Ndongo Gueye, Libasse Diop, Kieran C. Molloy, Gabrielle Kociok-Köhn

**Affiliations:** a Laboratoire de Chimie Minérale et Analytique, Département de Chimie, Faculté des Sciences et Techniques, Université Cheikh Anta Diop, Dakar, Senegal; b Department of Chemistry, University of Bath, Claverton Down, Bath BA2 7AY, England

## Abstract

The title compound, (C_14_H_16_N)[Sn(C_6_H_5_)_3_(C_2_O_2_)], was synthesised by allowing C_2_O_4_(Bz_2_NH_2_)_2_ (Bz = benzyl) to react with SnPh_3_Cl. The asymmetric unit is built up by four SnPh_3_C_2_O_4_ anions and four Bz_2_NH_2_ cations which are related by a pseudo-inversion centre. Each Sn^IV^ cation is five-coordinated by the three phenyl groups and two O atoms belonging to the chelating oxalate ligand; the coordination geometry is that of a distorted trigonal bipyramid. Anions and cations are linked through N—H⋯O hydrogen bonds into a layer structure parallel to (001). Moreover, the anion–cation pairs are associated by two bifurcated N—H⋯O hydrogen bonds, generating pseudo-dimers. One of the phenyl groups of one anion is disordered over two sets of sites in a 0.69:0.31 ratio. The Flack parameter value of 0.44 (1) indicates racemic twinning.

## Related literature

For related structures, see: Ng & Rae (2000 ▶); Ng & Hook (1999 ▶); Ng & Kumar Das (1995 ▶). For general background, see: Evans & Karpel (1985 ▶).
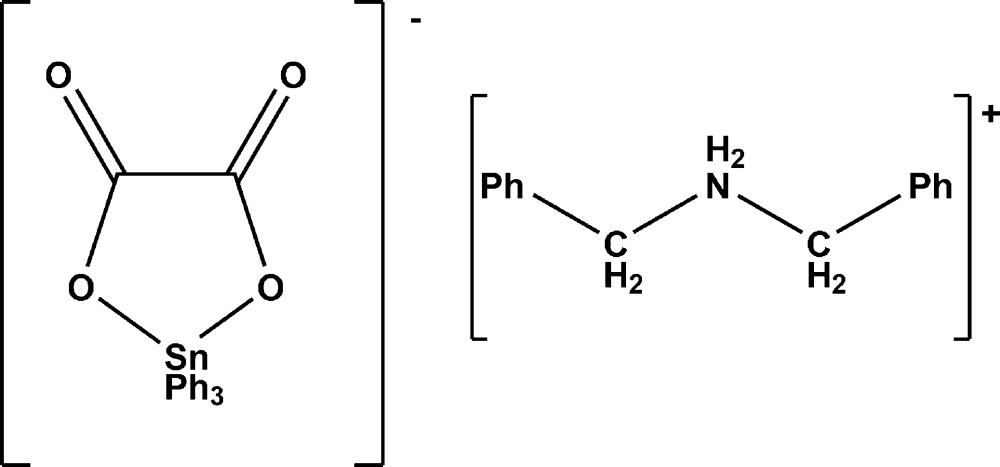


## Experimental

### 

#### Crystal data

(C_14_H_16_N)[Sn(C_6_H_5_)_3_(C_2_O_2_)]
*M* *_r_* = 636.31
Triclinic, 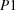

*a* = 9.8575 (2) Å
*b* = 16.8894 (3) Å
*c* = 18.9812 (4) Å
α = 110.444 (1)°
β = 96.082 (1)°
γ = 93.384 (1)°
*V* = 2928.73 (10) Å^3^
*Z* = 4
Mo *K*α radiation
μ = 0.91 mm^−1^
*T* = 150 K
0.40 × 0.20 × 0.10 mm

#### Data collection

Nonius KappaCCD diffractometer
Absorption correction: multi-scan (*SORTAV*; Blessing, 1997 ▶) *T*_min_ = 0.712, *T*_max_ = 0.914
56750 measured reflections
22478 independent reflections
20136 reflections with *I* > 2σ(*I*)
*R*_int_ = 0.038

#### Refinement

*R*[*F* ^2^ > 2σ(*F* ^2^)] = 0.031
*wR*(*F* ^2^) = 0.070
*S* = 1.06
22474 reflections
1424 parameters
15 restraints
H-atom parameters constrained
Δρ_max_ = 0.61 e Å^−3^
Δρ_min_ = −0.86 e Å^−3^
Absolute structure: Flack (1983 ▶), with 10508 Fridel pairs
Flack parameter: 0.438 (10)

### 

Data collection: *COLLECT* (Nonius, 1997 ▶); cell refinement: *SCALEPACK* (Otwinowski & Minor, 1997 ▶); data reduction: *SCALEPACK*; program(s) used to solve structure: *SIR97* (Altomare *et al.*, 1999 ▶); program(s) used to refine structure: *SHELXL97* (Sheldrick, 2008 ▶); molecular graphics: *ORTEPIII* (Burnett & Johnson, 1996 ▶), *ORTEP-3 for Windows* (Farrugia, 1997 ▶) and *PLATON* (Spek, 2009 ▶); software used to prepare material for publication: *SHELXL97*.

## Supplementary Material

Crystal structure: contains datablock(s) I, global. DOI: 10.1107/S1600536812021125/kp2394sup1.cif

Structure factors: contains datablock(s) I. DOI: 10.1107/S1600536812021125/kp2394Isup2.hkl

Additional supplementary materials:  crystallographic information; 3D view; checkCIF report

## Figures and Tables

**Table 1:** Selected bond lengths (Å)

Sn1—O1	2.124 (3)
Sn1—C121	2.131 (4)
Sn1—C111	2.156 (4)
Sn1—C131	2.160 (4)
Sn1—O2	2.306 (3)
Sn2—O6	2.116 (3)
Sn2—C221	2.131 (5)
Sn2—C211	2.147 (4)
Sn2—C231	2.167 (4)
Sn2—O5	2.317 (3)
Sn3—O9	2.104 (3)
Sn3—C311	2.142 (4)
Sn3—C321	2.142 (4)
Sn3—C331	2.185 (4)
Sn3—O10	2.355 (3)
Sn4—O13	2.117 (3)
Sn4—C421	2.133 (4)
Sn4—C411	2.140 (4)
Sn4—C431	2.168 (4)
Sn4—O14	2.289 (3)

**Table 2:** Hydrogen-bond geometry (Å, °)

*D*—H⋯*A*	*D*—H	H⋯*A*	*D*⋯*A*	*D*—H⋯*A*
N1—H1*A*⋯O4	0.92	1.94	2.847 (4)	171
N1—H1*B*⋯O15	0.92	2.26	2.902 (4)	126
N1—H1*B*⋯O16	0.92	1.98	2.805 (4)	148
N2—H2*A*⋯O7	0.92	1.92	2.795 (4)	159
N2—H2*B*⋯O11	0.92	2.05	2.857 (4)	146
N2—H2*B*⋯O12	0.92	2.27	2.925 (4)	128
N3—H3*A*⋯O7	0.92	2.21	2.951 (4)	137
N3—H3*A*⋯O8	0.92	2.08	2.813 (4)	136
N3—H3*B*⋯O11	0.92	1.91	2.816 (4)	169
N4—H4*A*⋯O3	0.92	2.15	2.843 (4)	131
N4—H4*A*⋯O4	0.92	2.10	2.843 (4)	137
N4—H4*B*⋯O16	0.92	1.84	2.755 (4)	177

**Fig. 1.**
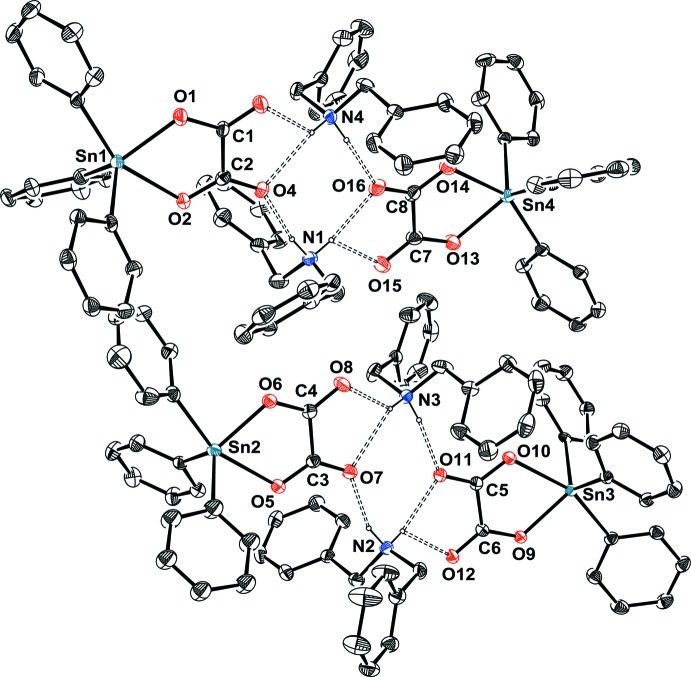
View of the unit cell with the atom numbering scheme. Ellipsoids are drawn at the 30% probability level. H atoms are drawn as small spheres of arbitrary radii. N—H···O hydrogen bonds are represented by dashed lines. H atoms attached to C atoms have been omitted for the sake of clarity.

## supplementary crystallographic information

### Comment

New organotin (IV) compounds are of interest because of their physical applications (Evans & Karpel, 1985). The X-ray structure of C_2_O_4_SnPh_3_Cy_2_NH_2_ were reported by (Ng & Kumar Das, 1995; Ng & Hook, 1999; Ng & Rae, 2000); it consists of two [C_2_O_4_SnPh_3_]^-^ anions connected into dimers by identical N—H—O hydrogen bonds. We have modified the counter ion by using Bz_2_NH_2_.

The asymmetric unit is build up by the association of four C_2_O_4_SnPh_3_ anions and four Bz_2_NH_2_ cations (Fig. 1, Table 1). In each anion, the oxalate ion chelates the SnPh_3_ residue. Sn atoms are in pentagonal environments. Anion and cation are linked by N—H···O hydrogen bonds. Moreover, anion and cation pairs are associated by two bifurcated N—H···O hydrogen bonds into a pseudo dimer (Table 2, Fig. 1). The type of dimers observed in the title compound is different from the one reported by Ng & Rae (2000). The Sn—O bonds (Table 1) indicate asymmetrical chelation.

### Experimental

When an ethanolic solution of (Bz_2_NH_2_)_2_C_2_O_4_ is mixed with a similar solution of SnPh_3_Cl in 1/2 ratio, crystals of Bz_2_NH_2_C_2_O_4_SnPh_3_ suitable for X-ray work were obtained after slow evaporation.

All the chemicals were from ALDRICH and used without any further purification.

### Refinement

All H atoms attached to C and N atoms were fixed geometrically and treated as riding with C—H = 0.98 Å (methyl) or 0.97 Å (methylene) and N—H = 0.86 Å with *U*_iso_(H) = 1.2*U*_eq_(C or N).

One of the phenyl group attached to Sn2 is disordered over two positions by rotation around the central C—C axis with occupancy factor in the ratio 0.69/0.31. The C—C distances and C—C—C angles were restrained to reasonable dimensions by using the SAME instruction available within *SHELXL97* (Sheldrick, 2008). The anistropic thermal parameters were severely restrained using the EADP instruction.

The value of the Flack's parameters is indicative of a twin by inversion (racemic twinning). Although, ADDSYM within *PLATON* (Spek, 2009) detects (pseudo) centre of symmetry, no model could be correctly refined in the centrosymmetric P1 space group.

### Crystal data

**Table:**

(C_14_H_16_N)[Sn(C_6_H_5_)_3_(C_2_O_2_)]	*Z* = 4
*M**_r_* = 636.31	*F*(000) = 1296
Triclinic, *P*1	*D*_x_ = 1.443 Mg m^−^^3^
Hall symbol: P 1	Mo *K*α radiation, λ = 0.71073 Å
*a* = 9.8575 (2) Å	Cell parameters from 70274 reflections
*b* = 16.8894 (3) Å	θ = 2.9–27.5°
*c* = 18.9812 (4) Å	µ = 0.91 mm^−^^1^
α = 110.444 (1)°	*T* = 150 K
β = 96.082 (1)°	Block, colourless
γ = 93.384 (1)°	0.40 × 0.20 × 0.10 mm
*V* = 2928.73 (10) Å^3^	

### Data collection

**Table:**

Nonius KappaCCD diffractometer	22478 independent reflections
Radiation source: fine-focus sealed tube	20136 reflections with *I* > 2σ(*I*)
Graphite monochromator	*R*_int_ = 0.038
305 2.0 degree images with φ and ω scans	θ_max_ = 26.4°, θ_min_ = 3.2°
Absorption correction: multi-scan (*SORTAV*; Blessing, 1997)	*h* = −12→12
*T*_min_ = 0.712, *T*_max_ = 0.914	*k* = −21→21
56750 measured reflections	*l* = −23→23

### Refinement

**Table:**

Refinement on *F*^2^	Secondary atom site location: difference Fourier map
Least-squares matrix: full	Hydrogen site location: inferred from neighbouring sites
*R*[*F*^2^ > 2σ(*F*^2^)] = 0.031	H-atom parameters constrained
*wR*(*F*^2^) = 0.070	*w* = 1/[σ^2^(*F*_o_^2^) + (0.0332*P*)^2^ + 0.514*P*] where *P* = (*F*_o_^2^ + 2*F*_c_^2^)/3
*S* = 1.06	(Δ/σ)_max_ = 0.007
22474 reflections	Δρ_max_ = 0.61 e Å^−^^3^
1424 parameters	Δρ_min_ = −0.86 e Å^−^^3^
15 restraints	Absolute structure: Flack (1983), with 10508 Fridel pairs
Primary atom site location: structure-invariant direct methods	Flack parameter: 0.438 (10)

### Special details

**Table:**

Geometry. All e.s.d.'s (except the e.s.d. in the dihedral angle between two l.s. planes) are estimated using the full covariance matrix. The cell e.s.d.'s are taken into account individually in the estimation of e.s.d.'s in distances, angles and torsion angles; correlations between e.s.d.'s in cell parameters are only used when they are defined by crystal symmetry. An approximate (isotropic) treatment of cell e.s.d.'s is used for estimating e.s.d.'s involving l.s. planes.
Refinement. Refinement of *F*^2^ against ALL reflections. The weighted *R*-factor *wR* and goodness of fit *S* are based on *F*^2^, conventional *R*-factors *R* are based on *F*, with *F* set to zero for negative *F*^2^. The threshold expression of *F*^2^ > σ(*F*^2^) is used only for calculating *R*-factors(gt) *etc*. and is not relevant to the choice of reflections for refinement. *R*-factors based on *F*^2^ are statistically about twice as large as those based on *F*, and *R*-factors based on ALL data will be even larger.

### Fractional atomic coordinates and isotropic or equivalent isotropic displacement parameters (Å^2^)

**Table:**

	*x*	*y*	*z*	*U*_iso_*/*U*_eq_	Occ. (<1)
Sn1	0.03511 (2)	0.316679 (15)	−0.230967 (14)	0.02958 (7)	
O1	0.0397 (4)	0.31295 (18)	−0.12006 (15)	0.0379 (8)	
O2	0.1278 (3)	0.44817 (18)	−0.14581 (15)	0.0324 (7)	
O3	0.0906 (3)	0.37964 (18)	0.00463 (15)	0.0409 (8)	
O4	0.1723 (3)	0.52393 (17)	−0.02096 (14)	0.0348 (7)	
C1	0.0845 (4)	0.3788 (3)	−0.0606 (2)	0.0295 (9)	
C2	0.1337 (4)	0.4575 (3)	−0.0767 (2)	0.0287 (9)	
C111	−0.0942 (4)	0.3882 (3)	−0.2797 (2)	0.0350 (10)	
C112	−0.1393 (5)	0.4646 (3)	−0.2382 (2)	0.0434 (11)	
H112	−0.1163	0.4872	−0.1846	0.052*	
C113	−0.2174 (6)	0.5085 (4)	−0.2740 (3)	0.0560 (14)	
H113	−0.2466	0.5614	−0.2452	0.067*	
C114	−0.2527 (5)	0.4753 (3)	−0.3514 (3)	0.0479 (13)	
H114	−0.3085	0.5048	−0.3757	0.058*	
C115	−0.2086 (5)	0.4003 (3)	−0.3938 (2)	0.0420 (11)	
H115	−0.2313	0.3784	−0.4475	0.050*	
C116	−0.1304 (5)	0.3568 (3)	−0.3578 (2)	0.0376 (10)	
H116	−0.1008	0.3042	−0.3871	0.045*	
C121	0.2246 (4)	0.2965 (3)	−0.2760 (2)	0.0313 (9)	
C122	0.3270 (4)	0.3617 (3)	−0.2628 (2)	0.0353 (9)	
H122	0.3184	0.4170	−0.2282	0.042*	
C123	0.4429 (4)	0.3470 (3)	−0.3001 (2)	0.0407 (10)	
H123	0.5114	0.3925	−0.2916	0.049*	
C124	0.4588 (4)	0.2668 (3)	−0.3493 (2)	0.0405 (10)	
H124	0.5378	0.2574	−0.3746	0.049*	
C125	0.3596 (5)	0.2003 (3)	−0.3617 (2)	0.0405 (11)	
H125	0.3708	0.1447	−0.3947	0.049*	
C126	0.2429 (4)	0.2153 (3)	−0.3252 (2)	0.0341 (9)	
H126	0.1746	0.1695	−0.3340	0.041*	
C131	−0.0762 (4)	0.1924 (3)	−0.2639 (2)	0.0351 (10)	
C132	−0.0249 (5)	0.1319 (3)	−0.2359 (3)	0.0454 (11)	
H132	0.0624	0.1451	−0.2058	0.054*	
C133	−0.0962 (6)	0.0543 (3)	−0.2504 (3)	0.0574 (14)	
H133	−0.0579	0.0141	−0.2315	0.069*	
C134	−0.2278 (6)	0.0351 (4)	−0.2941 (3)	0.0615 (16)	
H134	−0.2802	−0.0175	−0.3033	0.074*	
C135	−0.2790 (5)	0.0929 (4)	−0.3229 (3)	0.0606 (16)	
H135	−0.3660	0.0792	−0.3534	0.073*	
C136	−0.2054 (5)	0.1713 (3)	−0.3080 (2)	0.0446 (12)	
H136	−0.2433	0.2108	−0.3280	0.054*	
N1	0.2842 (3)	0.6970 (2)	0.03179 (18)	0.0317 (8)	
H1A	0.2495	0.6409	0.0095	0.038*	
H1B	0.2505	0.7189	0.0773	0.038*	
C141	0.2338 (4)	0.7433 (3)	−0.0179 (3)	0.0452 (11)	
H14A	0.2579	0.7150	−0.0695	0.054*	
H14B	0.2790	0.8022	0.0022	0.054*	
C142	0.0808 (5)	0.7450 (3)	−0.0220 (3)	0.0410 (11)	
C143	−0.0076 (4)	0.6755 (3)	−0.0680 (2)	0.0452 (10)	
H143	0.0287	0.6256	−0.0977	0.054*	
C144	−0.1499 (5)	0.6772 (3)	−0.0716 (3)	0.0511 (12)	
H144	−0.2097	0.6283	−0.1024	0.061*	
C145	−0.2023 (5)	0.7502 (3)	−0.0301 (3)	0.0467 (11)	
H145	−0.2987	0.7526	−0.0343	0.056*	
C146	−0.1171 (5)	0.8191 (3)	0.0168 (3)	0.0518 (12)	
H146	−0.1548	0.8685	0.0462	0.062*	
C147	0.0254 (5)	0.8178 (3)	0.0222 (3)	0.0474 (11)	
H147	0.0842	0.8658	0.0556	0.057*	
C151	0.4385 (4)	0.7016 (3)	0.0473 (2)	0.0379 (9)	
H15A	0.4801	0.7531	0.0410	0.045*	
H15B	0.4658	0.7062	0.1004	0.045*	
C152	0.4914 (4)	0.6244 (2)	−0.0056 (2)	0.0339 (9)	
C153	0.4914 (4)	0.6112 (3)	−0.0820 (2)	0.0389 (9)	
H153	0.4594	0.6525	−0.1014	0.047*	
C154	0.5374 (5)	0.5387 (3)	−0.1303 (3)	0.0484 (11)	
H154	0.5340	0.5294	−0.1828	0.058*	
C155	0.5891 (5)	0.4787 (3)	−0.1013 (3)	0.0522 (13)	
H155	0.6212	0.4293	−0.1345	0.063*	
C156	0.5939 (5)	0.4905 (3)	−0.0262 (3)	0.0462 (13)	
H156	0.6302	0.4502	−0.0067	0.055*	
C157	0.5434 (5)	0.5641 (3)	0.0222 (3)	0.0451 (11)	
H157	0.5449	0.5727	0.0745	0.054*	
Sn2	0.51794 (2)	0.821131 (14)	−0.203097 (14)	0.02842 (7)	
O5	0.6317 (3)	0.94752 (18)	−0.11733 (15)	0.0324 (7)	
O6	0.4827 (3)	0.82714 (17)	−0.09338 (15)	0.0313 (6)	
O7	0.6615 (3)	1.02853 (17)	0.00613 (15)	0.0350 (7)	
O8	0.5115 (3)	0.90160 (17)	0.03147 (15)	0.0393 (7)	
C3	0.6163 (4)	0.9635 (3)	−0.0487 (2)	0.0273 (9)	
C4	0.5301 (4)	0.8937 (2)	−0.0336 (2)	0.0293 (9)	
C211	0.4038 (4)	0.8984 (3)	−0.2516 (2)	0.0275 (9)	
C212	0.4369 (5)	0.9847 (3)	−0.2329 (2)	0.0366 (10)	
H212	0.5127	1.0135	−0.1955	0.044*	
C213	0.3593 (5)	1.0289 (3)	−0.2688 (2)	0.0434 (11)	
H213	0.3823	1.0882	−0.2553	0.052*	
C214	0.2509 (5)	0.9891 (3)	−0.3230 (3)	0.0454 (12)	
H214	0.1981	1.0204	−0.3466	0.055*	
C215	0.2183 (5)	0.9027 (3)	−0.3433 (3)	0.0472 (12)	
H215	0.1443	0.8740	−0.3821	0.057*	
C216	0.2929 (4)	0.8583 (3)	−0.3074 (2)	0.0376 (10)	
H216	0.2686	0.7992	−0.3209	0.045*	
C221	0.7107 (5)	0.7938 (3)	−0.2423 (3)	0.0529 (6)	
C222	0.8358 (6)	0.8395 (4)	−0.2051 (4)	0.0529 (6)	0.69
H222	0.8385	0.8827	−0.1566	0.063*	0.69
C223	0.9568 (7)	0.8237 (4)	−0.2366 (4)	0.0529 (6)	0.69
H223	1.0415	0.8554	−0.2107	0.063*	0.69
C224	0.9496 (5)	0.7584 (3)	−0.3092 (3)	0.0529 (6)	
H224	1.0305	0.7461	−0.3323	0.063*	
C225	0.8344 (7)	0.7162 (5)	−0.3435 (4)	0.0529 (6)	0.69
H225	0.8319	0.6715	−0.3910	0.063*	0.69
C226	0.7128 (7)	0.7346 (5)	−0.3127 (4)	0.0529 (6)	0.69
H226	0.6287	0.7049	−0.3416	0.063*	0.69
C22A	0.7841 (14)	0.8550 (8)	−0.2558 (9)	0.0529 (6)	0.31
H22A	0.7560	0.9107	−0.2402	0.063*	0.31
C23A	0.8994 (13)	0.8392 (8)	−0.2916 (9)	0.0529 (6)	0.31
H23A	0.9451	0.8822	−0.3046	0.063*	0.31
C25A	0.8550 (14)	0.6933 (9)	−0.3205 (9)	0.0529 (6)	0.31
H25A	0.8652	0.6376	−0.3537	0.063*	0.31
C26A	0.7398 (14)	0.7101 (8)	−0.2814 (9)	0.0529 (6)	0.31
H26A	0.6802	0.6638	−0.2814	0.063*	0.31
C232	0.4246 (5)	0.6248 (3)	−0.2878 (3)	0.0551 (12)	
H232	0.5137	0.6217	−0.3027	0.066*	
C233	0.3315 (7)	0.5507 (3)	−0.3122 (3)	0.0706 (16)	
H233	0.3584	0.4986	−0.3443	0.085*	
C231	0.3876 (5)	0.7020 (3)	−0.2424 (2)	0.0336 (10)	
C234	0.2061 (6)	0.5523 (3)	−0.2910 (3)	0.0587 (15)	
H234	0.1453	0.5017	−0.3070	0.070*	
C235	0.1663 (5)	0.6278 (3)	−0.2459 (3)	0.0580 (13)	
H235	0.0770	0.6298	−0.2312	0.070*	
C236	0.2559 (5)	0.7009 (3)	−0.2218 (2)	0.0480 (11)	
H236	0.2268	0.7524	−0.1901	0.058*	
N2	0.8261 (3)	1.1797 (2)	0.03425 (17)	0.0279 (7)	
H2A	0.7774	1.1269	0.0130	0.034*	
H2B	0.7827	1.2116	0.0736	0.034*	
C241	0.8219 (4)	1.2206 (3)	−0.0240 (2)	0.0364 (10)	
H24A	0.8607	1.1838	−0.0687	0.044*	
H24B	0.8804	1.2755	−0.0029	0.044*	
C242	0.6781 (4)	1.2363 (2)	−0.0495 (2)	0.0302 (9)	
C243	0.5821 (4)	1.2618 (3)	−0.0002 (2)	0.0385 (10)	
H243	0.6041	1.2674	0.0514	0.046*	
C244	0.4554 (4)	1.2789 (3)	−0.0252 (2)	0.0442 (10)	
H244	0.3900	1.2960	0.0092	0.053*	
C245	0.4223 (5)	1.2715 (3)	−0.1000 (2)	0.0425 (11)	
H245	0.3345	1.2831	−0.1174	0.051*	
C246	0.5183 (5)	1.2470 (3)	−0.1487 (2)	0.0435 (12)	
H246	0.4971	1.2425	−0.2001	0.052*	
C247	0.6455 (5)	1.2287 (3)	−0.1238 (2)	0.0367 (10)	
H247	0.7105	1.2109	−0.1582	0.044*	
C251	0.9683 (4)	1.1697 (3)	0.0660 (2)	0.0381 (10)	
H25B	1.0219	1.2263	0.0878	0.046*	
H25C	0.9614	1.1477	0.1076	0.046*	
C252	1.0434 (4)	1.1110 (3)	0.0078 (2)	0.0377 (10)	
C253	1.1379 (4)	1.1420 (3)	−0.0284 (2)	0.0411 (10)	
H253	1.1536	1.2014	−0.0172	0.049*	
C254	1.2092 (5)	1.0870 (3)	−0.0804 (3)	0.0487 (11)	
H254	1.2723	1.1084	−0.1055	0.058*	
C255	1.1883 (6)	1.0020 (3)	−0.0952 (3)	0.0640 (16)	
H255	1.2363	0.9639	−0.1312	0.077*	
C256	1.0987 (7)	0.9712 (3)	−0.0585 (4)	0.081 (2)	
H256	1.0865	0.9120	−0.0680	0.097*	
C257	1.0256 (6)	1.0260 (3)	−0.0074 (3)	0.0594 (15)	
H257	0.9625	1.0040	0.0173	0.071*	
Sn3	0.85092 (2)	1.406077 (13)	0.386037 (12)	0.02407 (6)	
O9	0.8803 (3)	1.40967 (17)	0.27899 (14)	0.0294 (6)	
O10	0.7281 (3)	1.28399 (18)	0.29295 (15)	0.0300 (7)	
O11	0.6933 (3)	1.21442 (17)	0.16696 (14)	0.0313 (6)	
O12	0.8453 (3)	1.34779 (18)	0.15305 (15)	0.0361 (7)	
C5	0.7414 (4)	1.2751 (2)	0.2254 (2)	0.0260 (9)	
C6	0.8298 (4)	1.3492 (2)	0.2162 (2)	0.0265 (9)	
C311	0.9559 (4)	1.3190 (3)	0.4257 (2)	0.0253 (8)	
C312	0.8974 (4)	1.2365 (2)	0.4124 (2)	0.0330 (9)	
H312	0.8123	1.2158	0.3805	0.040*	
C313	0.9627 (5)	1.1844 (3)	0.4456 (2)	0.0377 (10)	
H313	0.9221	1.1287	0.4364	0.045*	
C314	1.0868 (5)	1.2143 (3)	0.4920 (2)	0.0394 (10)	
H314	1.1318	1.1791	0.5146	0.047*	
C315	1.1453 (5)	1.2958 (3)	0.5053 (2)	0.0393 (10)	
H315	1.2302	1.3165	0.5374	0.047*	
C316	1.0811 (4)	1.3465 (3)	0.4724 (2)	0.0332 (9)	
H316	1.1233	1.4019	0.4817	0.040*	
C321	0.6560 (4)	1.4362 (2)	0.4228 (2)	0.0209 (7)	
C322	0.5410 (4)	1.3769 (2)	0.4038 (2)	0.0307 (8)	
H322	0.5453	1.3205	0.3702	0.037*	
C323	0.4195 (5)	1.3997 (3)	0.4336 (3)	0.0433 (10)	
H323	0.3418	1.3589	0.4208	0.052*	
C324	0.4128 (5)	1.4825 (3)	0.4821 (3)	0.0429 (10)	
H324	0.3296	1.4985	0.5015	0.052*	
C325	0.5265 (4)	1.5416 (3)	0.5023 (2)	0.0355 (9)	
H325	0.5213	1.5978	0.5361	0.043*	
C326	0.6476 (4)	1.5196 (2)	0.4737 (2)	0.0309 (8)	
H326	0.7254	1.5606	0.4882	0.037*	
C331	0.9827 (4)	1.5259 (2)	0.4361 (2)	0.0290 (9)	
C332	1.0369 (4)	1.5553 (2)	0.5134 (2)	0.0332 (9)	
H332	1.0088	1.5255	0.5444	0.040*	
C333	1.1308 (4)	1.6272 (2)	0.5454 (2)	0.0393 (9)	
H333	1.1689	1.6450	0.5972	0.047*	
C334	1.1684 (5)	1.6728 (3)	0.5014 (2)	0.0425 (10)	
H334	1.2325	1.7219	0.5229	0.051*	
C335	1.1121 (5)	1.6464 (3)	0.4251 (2)	0.0454 (11)	
H335	1.1366	1.6781	0.3949	0.054*	
C336	1.0212 (4)	1.5746 (2)	0.3939 (2)	0.0385 (9)	
H336	0.9833	1.5575	0.3421	0.046*	
N3	0.5244 (3)	1.0645 (2)	0.14328 (17)	0.0274 (7)	
H3A	0.5629	1.0264	0.1050	0.033*	
H3B	0.5728	1.1169	0.1557	0.033*	
C341	0.5384 (5)	1.0388 (3)	0.2101 (3)	0.0473 (11)	
H34A	0.4723	0.9884	0.2003	0.057*	
H34B	0.5141	1.0853	0.2542	0.057*	
C342	0.6799 (4)	1.0180 (2)	0.2298 (2)	0.0336 (9)	
C343	0.7847 (5)	1.0171 (3)	0.1869 (3)	0.0500 (11)	
H343	0.7694	1.0314	0.1427	0.060*	
C344	0.9129 (5)	0.9954 (3)	0.2080 (3)	0.0553 (12)	
H344	0.9844	0.9948	0.1780	0.066*	
C345	0.9365 (6)	0.9748 (3)	0.2718 (3)	0.0539 (13)	
H345	1.0239	0.9598	0.2859	0.065*	
C346	0.8325 (6)	0.9762 (3)	0.3150 (3)	0.0525 (14)	
H346	0.8486	0.9623	0.3594	0.063*	
C347	0.7045 (5)	0.9976 (3)	0.2945 (2)	0.0442 (11)	
H347	0.6335	0.9983	0.3248	0.053*	
C351	0.3787 (4)	1.0692 (3)	0.1144 (2)	0.0418 (11)	
H35A	0.3249	1.0141	0.1045	0.050*	
H35B	0.3763	1.0784	0.0656	0.050*	
C352	0.3116 (4)	1.1397 (3)	0.1693 (2)	0.0317 (9)	
C353	0.2290 (5)	1.1208 (3)	0.2162 (3)	0.0506 (12)	
H353	0.2166	1.0644	0.2156	0.061*	
C354	0.1631 (5)	1.1844 (4)	0.2648 (3)	0.0583 (14)	
H354	0.1056	1.1712	0.2971	0.070*	
C355	0.1815 (5)	1.2673 (3)	0.2660 (3)	0.0558 (14)	
H355	0.1363	1.3108	0.2988	0.067*	
C356	0.2653 (5)	1.2856 (3)	0.2195 (3)	0.0525 (12)	
H356	0.2785	1.3419	0.2197	0.063*	
C357	0.3301 (5)	1.2223 (3)	0.1724 (3)	0.0421 (11)	
H357	0.3896	1.2359	0.1411	0.050*	
Sn4	0.30006 (2)	0.902830 (14)	0.390075 (15)	0.02991 (7)	
O13	0.3061 (4)	0.91751 (18)	0.28409 (16)	0.0421 (8)	
O14	0.2218 (4)	0.77437 (19)	0.29757 (17)	0.0409 (8)	
O15	0.2848 (4)	0.85576 (19)	0.15774 (16)	0.0480 (8)	
O16	0.2088 (3)	0.70374 (18)	0.17199 (16)	0.0424 (8)	
C7	0.2770 (5)	0.8537 (3)	0.2211 (2)	0.0365 (10)	
C8	0.2312 (4)	0.7689 (3)	0.2306 (2)	0.0361 (10)	
C411	0.4296 (4)	0.8326 (3)	0.4386 (2)	0.0323 (9)	
C412	0.4817 (5)	0.7565 (3)	0.3978 (3)	0.0408 (11)	
H412	0.4566	0.7307	0.3445	0.049*	
C413	0.5695 (5)	0.7199 (3)	0.4359 (3)	0.0525 (13)	
H413	0.6062	0.6694	0.4079	0.063*	
C414	0.6053 (5)	0.7541 (3)	0.5128 (3)	0.0524 (13)	
H414	0.6661	0.7276	0.5377	0.063*	
C415	0.5516 (5)	0.8285 (3)	0.5546 (3)	0.0479 (12)	
H415	0.5743	0.8526	0.6082	0.057*	
C416	0.4648 (5)	0.8668 (3)	0.5165 (2)	0.0393 (10)	
H416	0.4288	0.9175	0.5446	0.047*	
C421	0.1064 (4)	0.9153 (2)	0.4312 (2)	0.0289 (9)	
C422	0.0038 (5)	0.8460 (3)	0.4094 (3)	0.0437 (11)	
H422	0.0136	0.7949	0.3693	0.052*	
C423	−0.1107 (5)	0.8526 (3)	0.4465 (3)	0.0519 (12)	
H423	−0.1795	0.8059	0.4318	0.062*	
C424	−0.1257 (5)	0.9276 (3)	0.5054 (3)	0.0478 (12)	
H424	−0.2038	0.9314	0.5313	0.057*	
C425	−0.0284 (5)	0.9955 (3)	0.5261 (2)	0.0415 (11)	
H425	−0.0403	1.0472	0.5652	0.050*	
C426	0.0887 (4)	0.9889 (3)	0.4896 (2)	0.0342 (9)	
H426	0.1572	1.0359	0.5052	0.041*	
C431	0.4099 (4)	1.0288 (3)	0.4305 (2)	0.0291 (9)	
C432	0.5422 (4)	1.0466 (3)	0.4709 (2)	0.0336 (9)	
H432	0.5808	1.0047	0.4871	0.040*	
C433	0.6181 (5)	1.1241 (3)	0.4876 (2)	0.0425 (11)	
H433	0.7080	1.1350	0.5147	0.051*	
C434	0.5614 (5)	1.1856 (3)	0.4643 (3)	0.0462 (12)	
H434	0.6126	1.2390	0.4756	0.055*	
C435	0.4312 (5)	1.1696 (3)	0.4251 (3)	0.0466 (11)	
H435	0.3928	1.2117	0.4090	0.056*	
C436	0.3563 (4)	1.0919 (3)	0.4090 (2)	0.0376 (10)	
H436	0.2658	1.0818	0.3826	0.045*	
N4	0.0827 (3)	0.5401 (2)	0.12048 (17)	0.0285 (7)	
H4A	0.1251	0.5104	0.0797	0.034*	
H4B	0.1225	0.5954	0.1389	0.034*	
C441	−0.0675 (4)	0.5394 (3)	0.0929 (2)	0.0357 (9)	
H44A	−0.0758	0.5462	0.0430	0.043*	
H44B	−0.1162	0.4836	0.0859	0.043*	
C442	−0.1352 (4)	0.6083 (3)	0.1467 (2)	0.0347 (9)	
C443	−0.1991 (5)	0.5956 (3)	0.2029 (3)	0.0459 (11)	
H443	−0.1988	0.5424	0.2094	0.055*	
C444	−0.2648 (6)	0.6601 (3)	0.2510 (3)	0.0612 (14)	
H444	−0.3070	0.6511	0.2907	0.073*	
C445	−0.2681 (6)	0.7352 (3)	0.2408 (3)	0.0619 (15)	
H445	−0.3148	0.7785	0.2726	0.074*	
C446	−0.2035 (5)	0.7496 (3)	0.1839 (3)	0.0569 (14)	
H446	−0.2049	0.8029	0.1774	0.068*	
C447	−0.1380 (5)	0.6865 (3)	0.1376 (3)	0.0448 (11)	
H447	−0.0939	0.6961	0.0988	0.054*	
C451	0.1085 (5)	0.5034 (3)	0.1800 (2)	0.0427 (11)	
H45A	0.0583	0.4459	0.1625	0.051*	
H45B	0.0721	0.5389	0.2262	0.051*	
C452	0.2580 (5)	0.4972 (3)	0.2000 (2)	0.0343 (10)	
C453	0.3424 (5)	0.4641 (3)	0.1453 (2)	0.0494 (11)	
H453	0.3079	0.4490	0.0931	0.059*	
C454	0.4784 (5)	0.4526 (3)	0.1662 (3)	0.0500 (12)	
H454	0.5361	0.4305	0.1284	0.060*	
C455	0.5284 (5)	0.4735 (3)	0.2417 (2)	0.0430 (10)	
H455	0.6200	0.4650	0.2562	0.052*	
C456	0.4440 (5)	0.5069 (3)	0.2962 (2)	0.0442 (10)	
H456	0.4783	0.5215	0.3484	0.053*	
C457	0.3104 (4)	0.5193 (2)	0.2759 (2)	0.0390 (9)	
H457	0.2541	0.5431	0.3141	0.047*	

### Atomic displacement parameters (Å^2^)

**Table:**

	*U*^11^	*U*^22^	*U*^33^	*U*^12^	*U*^13^	*U*^23^
Sn1	0.02638 (14)	0.03560 (15)	0.02377 (12)	0.00268 (11)	0.00207 (11)	0.00741 (11)
O1	0.051 (2)	0.0324 (16)	0.0241 (14)	−0.0054 (15)	0.0007 (14)	0.0058 (12)
O2	0.0372 (17)	0.0344 (15)	0.0231 (14)	−0.0007 (13)	0.0020 (12)	0.0086 (12)
O3	0.057 (2)	0.0345 (15)	0.0275 (14)	−0.0050 (14)	0.0032 (14)	0.0091 (12)
O4	0.0408 (16)	0.0277 (14)	0.0277 (13)	−0.0060 (12)	0.0033 (12)	0.0018 (12)
C1	0.029 (2)	0.031 (2)	0.0222 (18)	−0.0008 (17)	0.0004 (16)	0.0041 (16)
C2	0.0222 (19)	0.028 (2)	0.033 (2)	0.0018 (16)	0.0028 (18)	0.0083 (18)
C111	0.026 (2)	0.042 (3)	0.035 (2)	0.0016 (19)	0.0020 (19)	0.0133 (19)
C112	0.037 (2)	0.060 (3)	0.030 (2)	0.020 (2)	0.0055 (19)	0.010 (2)
C113	0.055 (3)	0.070 (3)	0.044 (3)	0.034 (3)	0.011 (2)	0.016 (2)
C114	0.040 (3)	0.067 (3)	0.047 (3)	0.021 (2)	0.005 (2)	0.032 (3)
C115	0.041 (3)	0.059 (3)	0.027 (2)	0.004 (2)	0.0038 (19)	0.018 (2)
C116	0.043 (2)	0.042 (2)	0.0263 (19)	0.006 (2)	0.0044 (19)	0.0109 (17)
C121	0.031 (2)	0.040 (2)	0.0234 (18)	0.0061 (18)	−0.0009 (17)	0.0120 (17)
C122	0.037 (2)	0.034 (2)	0.033 (2)	0.0027 (18)	0.0073 (18)	0.0088 (17)
C123	0.033 (2)	0.047 (3)	0.046 (2)	−0.0022 (19)	0.007 (2)	0.022 (2)
C124	0.031 (2)	0.058 (3)	0.032 (2)	0.010 (2)	0.0083 (18)	0.012 (2)
C125	0.038 (2)	0.048 (3)	0.031 (2)	0.013 (2)	0.0037 (19)	0.0075 (18)
C126	0.031 (2)	0.037 (2)	0.031 (2)	0.0062 (18)	0.0053 (18)	0.0073 (17)
C131	0.027 (2)	0.045 (3)	0.025 (2)	−0.0036 (19)	0.0055 (18)	0.0020 (18)
C132	0.041 (3)	0.046 (3)	0.044 (2)	−0.004 (2)	−0.001 (2)	0.013 (2)
C133	0.062 (3)	0.045 (3)	0.058 (3)	−0.013 (3)	0.004 (3)	0.013 (2)
C134	0.059 (3)	0.058 (3)	0.048 (3)	−0.029 (3)	0.008 (3)	0.000 (3)
C135	0.040 (3)	0.081 (4)	0.039 (3)	−0.024 (3)	0.002 (2)	0.001 (3)
C136	0.035 (2)	0.059 (3)	0.030 (2)	−0.006 (2)	0.003 (2)	0.005 (2)
N1	0.0311 (17)	0.0313 (17)	0.0300 (16)	−0.0017 (14)	0.0068 (14)	0.0075 (14)
C141	0.035 (2)	0.054 (3)	0.056 (3)	0.000 (2)	0.008 (2)	0.031 (2)
C142	0.038 (2)	0.044 (3)	0.049 (2)	0.002 (2)	0.010 (2)	0.026 (2)
C143	0.041 (2)	0.040 (2)	0.048 (2)	0.0052 (19)	0.005 (2)	0.009 (2)
C144	0.042 (3)	0.048 (3)	0.053 (3)	0.001 (2)	−0.002 (2)	0.008 (2)
C145	0.037 (2)	0.049 (3)	0.059 (3)	0.007 (2)	0.005 (2)	0.025 (2)
C146	0.050 (3)	0.037 (2)	0.071 (3)	0.010 (2)	0.014 (2)	0.021 (2)
C147	0.047 (3)	0.035 (2)	0.059 (3)	−0.001 (2)	0.003 (2)	0.018 (2)
C151	0.029 (2)	0.042 (2)	0.037 (2)	−0.0007 (17)	−0.0005 (17)	0.0093 (18)
C152	0.027 (2)	0.032 (2)	0.041 (2)	−0.0034 (17)	0.0006 (18)	0.0126 (18)
C153	0.038 (2)	0.039 (2)	0.040 (2)	0.0020 (18)	0.0037 (18)	0.0165 (18)
C154	0.048 (3)	0.051 (3)	0.042 (2)	0.004 (2)	0.010 (2)	0.010 (2)
C155	0.042 (3)	0.035 (2)	0.071 (3)	0.001 (2)	0.013 (3)	0.006 (2)
C156	0.047 (3)	0.034 (2)	0.062 (3)	0.006 (2)	0.006 (3)	0.022 (2)
C157	0.039 (2)	0.050 (3)	0.043 (2)	−0.009 (2)	−0.005 (2)	0.019 (2)
Sn2	0.02770 (14)	0.02902 (15)	0.02921 (13)	0.00306 (11)	0.00688 (12)	0.01037 (11)
O5	0.0348 (16)	0.0350 (16)	0.0269 (14)	−0.0008 (13)	0.0075 (13)	0.0100 (12)
O6	0.0370 (16)	0.0257 (14)	0.0291 (14)	−0.0022 (12)	0.0073 (13)	0.0073 (11)
O7	0.0425 (16)	0.0300 (15)	0.0313 (14)	−0.0011 (12)	0.0097 (13)	0.0091 (12)
O8	0.0566 (19)	0.0314 (15)	0.0297 (15)	−0.0025 (13)	0.0162 (14)	0.0089 (12)
C3	0.0245 (19)	0.027 (2)	0.030 (2)	0.0034 (16)	0.0047 (17)	0.0097 (17)
C4	0.030 (2)	0.026 (2)	0.032 (2)	0.0051 (16)	0.0067 (17)	0.0089 (16)
C211	0.026 (2)	0.031 (2)	0.0267 (18)	0.0046 (17)	0.0074 (17)	0.0095 (16)
C212	0.038 (2)	0.038 (2)	0.034 (2)	0.0018 (19)	0.0066 (18)	0.0140 (18)
C213	0.055 (3)	0.029 (2)	0.044 (2)	0.002 (2)	0.000 (2)	0.0123 (18)
C214	0.050 (3)	0.042 (3)	0.045 (2)	0.017 (2)	0.002 (2)	0.017 (2)
C215	0.038 (2)	0.047 (3)	0.050 (3)	0.001 (2)	−0.007 (2)	0.014 (2)
C216	0.037 (2)	0.032 (2)	0.041 (2)	0.0034 (18)	0.0010 (19)	0.0113 (18)
C221	0.0361 (12)	0.0527 (14)	0.0569 (15)	0.0060 (10)	0.0147 (11)	0.0008 (11)
C222	0.0361 (12)	0.0527 (14)	0.0569 (15)	0.0060 (10)	0.0147 (11)	0.0008 (11)
C223	0.0361 (12)	0.0527 (14)	0.0569 (15)	0.0060 (10)	0.0147 (11)	0.0008 (11)
C224	0.0361 (12)	0.0527 (14)	0.0569 (15)	0.0060 (10)	0.0147 (11)	0.0008 (11)
C225	0.0361 (12)	0.0527 (14)	0.0569 (15)	0.0060 (10)	0.0147 (11)	0.0008 (11)
C226	0.0361 (12)	0.0527 (14)	0.0569 (15)	0.0060 (10)	0.0147 (11)	0.0008 (11)
C22A	0.0361 (12)	0.0527 (14)	0.0569 (15)	0.0060 (10)	0.0147 (11)	0.0008 (11)
C23A	0.0361 (12)	0.0527 (14)	0.0569 (15)	0.0060 (10)	0.0147 (11)	0.0008 (11)
C25A	0.0361 (12)	0.0527 (14)	0.0569 (15)	0.0060 (10)	0.0147 (11)	0.0008 (11)
C26A	0.0361 (12)	0.0527 (14)	0.0569 (15)	0.0060 (10)	0.0147 (11)	0.0008 (11)
C232	0.066 (3)	0.031 (2)	0.069 (3)	0.003 (2)	0.027 (3)	0.015 (2)
C233	0.100 (5)	0.032 (2)	0.075 (4)	0.002 (3)	0.016 (3)	0.013 (2)
C231	0.040 (2)	0.032 (2)	0.030 (2)	0.0033 (18)	0.0028 (19)	0.0133 (17)
C234	0.068 (4)	0.055 (3)	0.055 (3)	−0.025 (3)	−0.010 (3)	0.032 (3)
C235	0.048 (3)	0.074 (3)	0.047 (3)	−0.024 (3)	0.000 (2)	0.021 (3)
C236	0.045 (3)	0.053 (3)	0.038 (2)	−0.009 (2)	0.007 (2)	0.008 (2)
N2	0.0278 (17)	0.0279 (17)	0.0255 (15)	0.0013 (13)	0.0059 (14)	0.0059 (13)
C241	0.033 (2)	0.043 (2)	0.037 (2)	0.0008 (18)	0.0066 (18)	0.0201 (19)
C242	0.031 (2)	0.030 (2)	0.0317 (19)	−0.0006 (17)	0.0028 (17)	0.0140 (16)
C243	0.035 (2)	0.052 (3)	0.033 (2)	0.005 (2)	0.0061 (18)	0.0195 (19)
C244	0.035 (2)	0.055 (3)	0.043 (2)	0.008 (2)	0.0056 (19)	0.017 (2)
C245	0.037 (2)	0.041 (2)	0.045 (2)	0.0022 (19)	−0.005 (2)	0.0127 (19)
C246	0.057 (3)	0.042 (3)	0.028 (2)	0.001 (2)	−0.007 (2)	0.0121 (19)
C247	0.045 (3)	0.037 (2)	0.030 (2)	0.006 (2)	0.0054 (19)	0.0140 (18)
C251	0.031 (2)	0.052 (3)	0.029 (2)	0.005 (2)	−0.0001 (18)	0.0123 (19)
C252	0.029 (2)	0.045 (3)	0.044 (2)	0.0101 (18)	0.0057 (19)	0.021 (2)
C253	0.033 (2)	0.049 (3)	0.047 (2)	0.005 (2)	0.006 (2)	0.024 (2)
C254	0.040 (2)	0.063 (3)	0.053 (3)	0.009 (2)	0.019 (2)	0.029 (2)
C255	0.068 (4)	0.055 (3)	0.082 (4)	0.031 (3)	0.049 (3)	0.025 (3)
C256	0.095 (5)	0.037 (3)	0.129 (6)	0.023 (3)	0.070 (5)	0.035 (3)
C257	0.062 (3)	0.043 (3)	0.088 (4)	0.016 (2)	0.045 (3)	0.029 (3)
Sn3	0.02328 (13)	0.02548 (13)	0.02308 (12)	0.00236 (11)	0.00516 (11)	0.00767 (10)
O9	0.0319 (15)	0.0308 (14)	0.0229 (13)	−0.0046 (12)	0.0051 (12)	0.0071 (11)
O10	0.0333 (16)	0.0297 (15)	0.0263 (14)	−0.0007 (12)	0.0063 (13)	0.0093 (12)
O11	0.0357 (15)	0.0290 (14)	0.0250 (13)	−0.0041 (12)	0.0038 (12)	0.0057 (11)
O12	0.0453 (17)	0.0356 (15)	0.0239 (14)	−0.0067 (13)	0.0061 (13)	0.0076 (11)
C5	0.0241 (19)	0.027 (2)	0.0263 (19)	0.0062 (16)	0.0049 (17)	0.0073 (16)
C6	0.028 (2)	0.027 (2)	0.0249 (19)	0.0043 (16)	0.0064 (17)	0.0099 (16)
C311	0.0245 (19)	0.029 (2)	0.0234 (17)	0.0073 (16)	0.0071 (16)	0.0083 (15)
C312	0.033 (2)	0.033 (2)	0.0332 (19)	0.0033 (18)	0.0021 (18)	0.0127 (17)
C313	0.043 (2)	0.030 (2)	0.043 (2)	0.0042 (18)	0.007 (2)	0.0172 (18)
C314	0.043 (3)	0.039 (2)	0.041 (2)	0.016 (2)	0.008 (2)	0.0174 (19)
C315	0.031 (2)	0.047 (3)	0.040 (2)	0.006 (2)	−0.0011 (19)	0.018 (2)
C316	0.026 (2)	0.035 (2)	0.037 (2)	0.0014 (17)	0.0050 (17)	0.0107 (17)
C321	0.0238 (18)	0.0219 (17)	0.0244 (17)	0.0082 (14)	0.0082 (15)	0.0151 (14)
C322	0.0274 (19)	0.0282 (19)	0.039 (2)	0.0028 (16)	0.0091 (17)	0.0134 (16)
C323	0.034 (2)	0.040 (2)	0.053 (3)	−0.0002 (19)	0.011 (2)	0.013 (2)
C324	0.032 (2)	0.045 (2)	0.049 (2)	0.0099 (19)	0.0146 (19)	0.010 (2)
C325	0.038 (2)	0.033 (2)	0.033 (2)	0.0072 (18)	0.0087 (18)	0.0074 (17)
C326	0.030 (2)	0.032 (2)	0.0269 (18)	0.0018 (16)	0.0000 (16)	0.0076 (16)
C331	0.026 (2)	0.028 (2)	0.031 (2)	0.0018 (16)	0.0056 (17)	0.0066 (16)
C332	0.036 (2)	0.032 (2)	0.0304 (19)	0.0030 (17)	0.0093 (17)	0.0091 (16)
C333	0.036 (2)	0.036 (2)	0.035 (2)	−0.0007 (18)	0.0024 (18)	0.0007 (17)
C334	0.040 (2)	0.028 (2)	0.047 (2)	−0.0042 (18)	0.008 (2)	−0.0010 (18)
C335	0.061 (3)	0.031 (2)	0.045 (2)	−0.004 (2)	0.018 (2)	0.0136 (19)
C336	0.052 (3)	0.030 (2)	0.0306 (19)	0.0014 (18)	0.0085 (18)	0.0070 (16)
N3	0.0249 (16)	0.0254 (16)	0.0272 (15)	0.0026 (13)	0.0047 (13)	0.0032 (12)
C341	0.038 (2)	0.059 (3)	0.066 (3)	0.012 (2)	0.021 (2)	0.044 (2)
C342	0.034 (2)	0.031 (2)	0.040 (2)	0.0090 (17)	0.0099 (18)	0.0166 (17)
C343	0.038 (2)	0.072 (3)	0.052 (3)	0.014 (2)	0.013 (2)	0.035 (2)
C344	0.039 (3)	0.073 (3)	0.052 (3)	0.014 (2)	0.009 (2)	0.019 (3)
C345	0.052 (3)	0.043 (3)	0.056 (3)	0.010 (2)	−0.014 (3)	0.010 (2)
C346	0.068 (4)	0.044 (3)	0.041 (2)	0.010 (3)	−0.010 (3)	0.014 (2)
C347	0.056 (3)	0.041 (2)	0.037 (2)	0.008 (2)	0.013 (2)	0.0137 (19)
C351	0.027 (2)	0.043 (2)	0.041 (2)	0.0062 (19)	−0.0026 (19)	−0.0010 (19)
C352	0.027 (2)	0.037 (2)	0.0289 (19)	0.0054 (17)	0.0033 (16)	0.0078 (17)
C353	0.040 (2)	0.054 (3)	0.070 (3)	0.011 (2)	0.015 (2)	0.034 (2)
C354	0.047 (3)	0.084 (4)	0.061 (3)	0.027 (3)	0.027 (3)	0.039 (3)
C355	0.045 (3)	0.065 (3)	0.044 (3)	0.024 (3)	0.000 (2)	0.002 (2)
C356	0.046 (3)	0.032 (2)	0.074 (3)	0.008 (2)	0.009 (3)	0.011 (2)
C357	0.037 (2)	0.048 (3)	0.046 (2)	0.002 (2)	0.004 (2)	0.023 (2)
Sn4	0.02951 (14)	0.02650 (14)	0.03272 (14)	0.00071 (11)	0.00739 (12)	0.00882 (11)
O13	0.061 (2)	0.0285 (15)	0.0313 (15)	−0.0081 (15)	0.0126 (15)	0.0045 (12)
O14	0.055 (2)	0.0302 (16)	0.0348 (16)	−0.0071 (14)	0.0100 (16)	0.0087 (13)
O15	0.067 (2)	0.0410 (17)	0.0344 (16)	−0.0081 (16)	0.0116 (15)	0.0124 (13)
O16	0.0494 (18)	0.0284 (14)	0.0388 (15)	−0.0083 (13)	0.0076 (14)	0.0006 (12)
C7	0.041 (2)	0.030 (2)	0.039 (2)	−0.0017 (18)	0.009 (2)	0.0119 (18)
C8	0.032 (2)	0.033 (2)	0.037 (2)	−0.0011 (18)	0.0041 (19)	0.0050 (19)
C411	0.030 (2)	0.033 (2)	0.036 (2)	0.0035 (18)	0.0121 (19)	0.0132 (17)
C412	0.044 (3)	0.037 (2)	0.046 (2)	0.012 (2)	0.013 (2)	0.017 (2)
C413	0.052 (3)	0.039 (3)	0.074 (3)	0.016 (2)	0.021 (3)	0.024 (2)
C414	0.042 (3)	0.052 (3)	0.075 (3)	0.001 (2)	0.006 (3)	0.038 (3)
C415	0.052 (3)	0.049 (3)	0.046 (2)	−0.009 (2)	−0.002 (2)	0.027 (2)
C416	0.042 (2)	0.032 (2)	0.043 (2)	0.0019 (19)	0.006 (2)	0.0121 (18)
C421	0.0237 (19)	0.035 (2)	0.0318 (19)	0.0000 (17)	0.0010 (16)	0.0183 (17)
C422	0.039 (2)	0.039 (2)	0.051 (3)	−0.003 (2)	0.008 (2)	0.014 (2)
C423	0.034 (2)	0.059 (3)	0.066 (3)	−0.007 (2)	0.012 (2)	0.026 (3)
C424	0.031 (2)	0.071 (3)	0.054 (3)	0.010 (2)	0.012 (2)	0.034 (3)
C425	0.039 (2)	0.053 (3)	0.034 (2)	0.016 (2)	0.0063 (19)	0.016 (2)
C426	0.031 (2)	0.041 (2)	0.033 (2)	0.0041 (18)	0.0000 (18)	0.0176 (18)
C431	0.029 (2)	0.031 (2)	0.0262 (19)	0.0016 (17)	0.0080 (17)	0.0086 (16)
C432	0.035 (2)	0.036 (2)	0.0297 (19)	0.0021 (18)	0.0081 (18)	0.0110 (17)
C433	0.041 (2)	0.049 (3)	0.032 (2)	−0.007 (2)	0.0022 (19)	0.0097 (19)
C434	0.054 (3)	0.036 (2)	0.038 (2)	−0.017 (2)	0.011 (2)	0.003 (2)
C435	0.058 (3)	0.032 (2)	0.051 (3)	0.002 (2)	0.011 (2)	0.015 (2)
C436	0.033 (2)	0.033 (2)	0.044 (2)	−0.0019 (18)	0.0044 (19)	0.0119 (18)
N4	0.0275 (17)	0.0308 (17)	0.0243 (15)	0.0018 (13)	0.0042 (14)	0.0061 (13)
C441	0.029 (2)	0.043 (2)	0.0294 (19)	0.0025 (17)	−0.0028 (17)	0.0073 (17)
C442	0.028 (2)	0.037 (2)	0.035 (2)	0.0001 (17)	−0.0006 (18)	0.0105 (18)
C443	0.048 (3)	0.043 (2)	0.053 (3)	0.004 (2)	0.016 (2)	0.021 (2)
C444	0.067 (3)	0.066 (3)	0.057 (3)	0.013 (3)	0.035 (3)	0.022 (3)
C445	0.059 (3)	0.049 (3)	0.062 (3)	0.006 (3)	0.015 (3)	0.000 (3)
C446	0.044 (3)	0.046 (3)	0.077 (4)	0.003 (2)	0.010 (3)	0.016 (3)
C447	0.038 (3)	0.046 (3)	0.049 (3)	0.006 (2)	0.003 (2)	0.016 (2)
C451	0.039 (2)	0.058 (3)	0.040 (2)	−0.003 (2)	0.009 (2)	0.029 (2)
C452	0.038 (2)	0.035 (2)	0.034 (2)	0.0000 (18)	0.0043 (19)	0.0183 (18)
C453	0.041 (2)	0.073 (3)	0.030 (2)	0.001 (2)	0.0030 (19)	0.015 (2)
C454	0.035 (2)	0.073 (3)	0.040 (2)	0.011 (2)	0.010 (2)	0.016 (2)
C455	0.036 (2)	0.051 (3)	0.047 (2)	0.010 (2)	0.002 (2)	0.024 (2)
C456	0.047 (3)	0.048 (2)	0.036 (2)	0.007 (2)	−0.008 (2)	0.0165 (19)
C457	0.046 (2)	0.039 (2)	0.0310 (19)	0.0075 (18)	0.0058 (18)	0.0097 (17)

### Geometric parameters (Å, º)

**Table:**

Sn1—O1	2.124 (3)	C255—C256	1.365 (8)
Sn1—C121	2.131 (4)	C255—H255	0.9500
Sn1—C111	2.156 (4)	C256—C257	1.384 (8)
Sn1—C131	2.160 (4)	C256—H256	0.9500
Sn1—O2	2.306 (3)	C257—H257	0.9500
O1—C1	1.290 (5)	Sn3—O9	2.104 (3)
O2—C2	1.260 (5)	Sn3—C311	2.142 (4)
O3—C1	1.228 (5)	Sn3—C321	2.142 (4)
O4—C2	1.248 (5)	Sn3—C331	2.185 (4)
C1—C2	1.528 (6)	Sn3—O10	2.355 (3)
C111—C112	1.384 (6)	O9—C6	1.295 (5)
C111—C116	1.387 (5)	O10—C5	1.259 (5)
C112—C113	1.383 (7)	O11—C5	1.242 (4)
C112—H112	0.9500	O12—C6	1.216 (5)
C113—C114	1.374 (7)	C5—C6	1.554 (6)
C113—H113	0.9500	C311—C316	1.392 (5)
C114—C115	1.366 (7)	C311—C312	1.402 (5)
C114—H114	0.9500	C312—C313	1.399 (6)
C115—C116	1.383 (6)	C312—H312	0.9500
C115—H115	0.9500	C313—C314	1.383 (6)
C116—H116	0.9500	C313—H313	0.9500
C121—C122	1.385 (6)	C314—C315	1.386 (6)
C121—C126	1.399 (6)	C314—H314	0.9500
C122—C123	1.396 (6)	C315—C316	1.371 (6)
C122—H122	0.9500	C315—H315	0.9500
C123—C124	1.379 (6)	C316—H316	0.9500
C123—H123	0.9500	C321—C322	1.395 (5)
C124—C125	1.383 (6)	C321—C326	1.416 (5)
C124—H124	0.9500	C322—C323	1.396 (6)
C125—C126	1.395 (6)	C322—H322	0.9500
C125—H125	0.9500	C323—C324	1.390 (6)
C126—H126	0.9500	C323—H323	0.9500
C131—C136	1.401 (6)	C324—C325	1.381 (6)
C131—C132	1.402 (7)	C324—H324	0.9500
C132—C133	1.373 (6)	C325—C326	1.380 (6)
C132—H132	0.9500	C325—H325	0.9500
C133—C134	1.417 (8)	C326—H326	0.9500
C133—H133	0.9500	C331—C336	1.396 (6)
C134—C135	1.371 (8)	C331—C332	1.407 (5)
C134—H134	0.9500	C332—C333	1.390 (5)
C135—C136	1.394 (7)	C332—H332	0.9500
C135—H135	0.9500	C333—C334	1.381 (6)
C136—H136	0.9500	C333—H333	0.9500
N1—C141	1.486 (5)	C334—C335	1.398 (6)
N1—C151	1.510 (5)	C334—H334	0.9500
N1—H1A	0.9200	C335—C336	1.372 (6)
N1—H1B	0.9200	C335—H335	0.9500
C141—C142	1.504 (6)	C336—H336	0.9500
C141—H14A	0.9900	N3—C341	1.474 (5)
C141—H14B	0.9900	N3—C351	1.499 (5)
C142—C143	1.380 (6)	N3—H3A	0.9200
C142—C147	1.403 (6)	N3—H3B	0.9200
C143—C144	1.399 (6)	C341—C342	1.500 (6)
C143—H143	0.9500	C341—H34A	0.9900
C144—C145	1.374 (6)	C341—H34B	0.9900
C144—H144	0.9500	C342—C343	1.379 (6)
C145—C146	1.362 (6)	C342—C347	1.388 (6)
C145—H145	0.9500	C343—C344	1.393 (6)
C146—C147	1.399 (6)	C343—H343	0.9500
C146—H146	0.9500	C344—C345	1.373 (7)
C147—H147	0.9500	C344—H344	0.9500
C151—C152	1.503 (6)	C345—C346	1.376 (8)
C151—H15A	0.9900	C345—H345	0.9500
C151—H15B	0.9900	C346—C347	1.387 (7)
C152—C153	1.387 (6)	C346—H346	0.9500
C152—C157	1.400 (6)	C347—H347	0.9500
C153—C154	1.384 (6)	C351—C352	1.522 (6)
C153—H153	0.9500	C351—H35A	0.9900
C154—C155	1.407 (7)	C351—H35B	0.9900
C154—H154	0.9500	C352—C353	1.370 (6)
C155—C156	1.364 (7)	C352—C357	1.375 (6)
C155—H155	0.9500	C353—C354	1.394 (7)
C156—C157	1.417 (7)	C353—H353	0.9500
C156—H156	0.9500	C354—C355	1.393 (8)
C157—H157	0.9500	C354—H354	0.9500
Sn2—O6	2.116 (3)	C355—C356	1.368 (7)
Sn2—C221	2.131 (5)	C355—H355	0.9500
Sn2—C211	2.147 (4)	C356—C357	1.373 (7)
Sn2—C231	2.167 (4)	C356—H356	0.9500
Sn2—O5	2.317 (3)	C357—H357	0.9500
O5—C3	1.262 (5)	Sn4—O13	2.117 (3)
O6—C4	1.305 (5)	Sn4—C421	2.133 (4)
O7—C3	1.238 (5)	Sn4—C411	2.140 (4)
O8—C4	1.230 (5)	Sn4—C431	2.168 (4)
C3—C4	1.538 (5)	Sn4—O14	2.289 (3)
C211—C212	1.383 (6)	O13—C7	1.290 (5)
C211—C216	1.393 (5)	O14—C8	1.256 (5)
C212—C213	1.386 (6)	O15—C7	1.226 (5)
C212—H212	0.9500	O16—C8	1.249 (5)
C213—C214	1.360 (6)	C7—C8	1.552 (6)
C213—H213	0.9500	C411—C416	1.382 (6)
C214—C215	1.381 (6)	C411—C412	1.407 (6)
C214—H214	0.9500	C412—C413	1.378 (7)
C215—C216	1.372 (6)	C412—H412	0.9500
C215—H215	0.9500	C413—C414	1.365 (7)
C216—H216	0.9500	C413—H413	0.9500
C221—C22A	1.339 (13)	C414—C415	1.401 (8)
C221—C226	1.364 (8)	C414—H414	0.9500
C221—C222	1.392 (8)	C415—C416	1.390 (6)
C221—C26A	1.411 (13)	C415—H415	0.9500
C222—C223	1.389 (9)	C416—H416	0.9500
C222—H222	0.9500	C421—C426	1.382 (6)
C223—C224	1.423 (8)	C421—C422	1.413 (5)
C223—H223	0.9500	C422—C423	1.383 (7)
C224—C225	1.283 (8)	C422—H422	0.9500
C224—C25A	1.341 (13)	C423—C424	1.394 (7)
C224—C23A	1.419 (13)	C423—H423	0.9500
C224—H224	0.9500	C424—C425	1.367 (7)
C225—C226	1.392 (9)	C424—H424	0.9500
C225—H225	0.9500	C425—C426	1.398 (6)
C226—H226	0.9500	C425—H425	0.9500
C22A—C23A	1.377 (15)	C426—H426	0.9500
C22A—H22A	0.9500	C431—C436	1.380 (6)
C23A—H23A	0.9500	C431—C432	1.400 (6)
C25A—C26A	1.410 (14)	C432—C433	1.384 (6)
C25A—H25A	0.9500	C432—H432	0.9500
C26A—H26A	0.9500	C433—C434	1.386 (7)
C232—C231	1.383 (6)	C433—H433	0.9500
C232—C233	1.412 (7)	C434—C435	1.375 (7)
C232—H232	0.9500	C434—H434	0.9500
C233—C234	1.339 (8)	C435—C436	1.385 (6)
C233—H233	0.9500	C435—H435	0.9500
C231—C236	1.396 (6)	C436—H436	0.9500
C234—C235	1.371 (7)	N4—C451	1.475 (5)
C234—H234	0.9500	N4—C441	1.515 (5)
C235—C236	1.379 (6)	N4—H4A	0.9200
C235—H235	0.9500	N4—H4B	0.9200
C236—H236	0.9500	C441—C442	1.499 (6)
N2—C241	1.493 (5)	C441—H44A	0.9900
N2—C251	1.508 (5)	C441—H44B	0.9900
N2—H2A	0.9200	C442—C443	1.366 (6)
N2—H2B	0.9200	C442—C447	1.392 (6)
C241—C242	1.518 (6)	C443—C444	1.397 (6)
C241—H24A	0.9900	C443—H443	0.9500
C241—H24B	0.9900	C444—C445	1.350 (7)
C242—C247	1.371 (5)	C444—H444	0.9500
C242—C243	1.383 (6)	C445—C446	1.393 (8)
C243—C244	1.372 (6)	C445—H445	0.9500
C243—H243	0.9500	C446—C447	1.369 (7)
C244—C245	1.382 (6)	C446—H446	0.9500
C244—H244	0.9500	C447—H447	0.9500
C245—C246	1.374 (7)	C451—C452	1.501 (6)
C245—H245	0.9500	C451—H45A	0.9900
C246—C247	1.383 (6)	C451—H45B	0.9900
C246—H246	0.9500	C452—C453	1.384 (6)
C247—H247	0.9500	C452—C457	1.388 (6)
C251—C252	1.499 (6)	C453—C454	1.402 (6)
C251—H25B	0.9900	C453—H453	0.9500
C251—H25C	0.9900	C454—C455	1.377 (6)
C252—C257	1.359 (6)	C454—H454	0.9500
C252—C253	1.393 (6)	C455—C456	1.382 (6)
C253—C254	1.383 (6)	C455—H455	0.9500
C253—H253	0.9500	C456—C457	1.382 (6)
C254—C255	1.362 (7)	C456—H456	0.9500
C254—H254	0.9500	C457—H457	0.9500
			
O1—Sn1—C121	114.33 (14)	C252—C253—H253	119.7
O1—Sn1—C111	126.26 (15)	C255—C254—C253	119.5 (5)
C121—Sn1—C111	113.62 (16)	C255—C254—H254	120.3
O1—Sn1—C131	83.18 (14)	C253—C254—H254	120.3
C121—Sn1—C131	105.98 (16)	C254—C255—C256	120.3 (5)
C111—Sn1—C131	105.50 (16)	C254—C255—H255	119.9
O1—Sn1—O2	72.26 (10)	C256—C255—H255	119.9
C121—Sn1—O2	90.45 (13)	C255—C256—C257	120.3 (5)
C111—Sn1—O2	84.60 (13)	C255—C256—H256	119.9
C131—Sn1—O2	154.56 (13)	C257—C256—H256	119.9
C1—O1—Sn1	121.4 (3)	C252—C257—C256	120.6 (5)
C2—O2—Sn1	115.8 (3)	C252—C257—H257	119.7
O3—C1—O1	123.9 (4)	C256—C257—H257	119.7
O3—C1—C2	121.1 (3)	O9—Sn3—C311	118.60 (13)
O1—C1—C2	114.9 (3)	O9—Sn3—C321	116.83 (12)
O4—C2—O2	127.1 (4)	C311—Sn3—C321	117.37 (14)
O4—C2—C1	117.4 (4)	O9—Sn3—C331	87.55 (13)
O2—C2—C1	115.6 (3)	C311—Sn3—C331	104.59 (15)
C112—C111—C116	118.2 (4)	C321—Sn3—C331	104.57 (15)
C112—C111—Sn1	124.1 (3)	O9—Sn3—O10	72.12 (10)
C116—C111—Sn1	117.7 (3)	C311—Sn3—O10	85.51 (12)
C113—C112—C111	120.7 (4)	C321—Sn3—O10	85.53 (12)
C113—C112—H112	119.7	C331—Sn3—O10	159.67 (12)
C111—C112—H112	119.7	C6—O9—Sn3	122.6 (2)
C114—C113—C112	119.8 (5)	C5—O10—Sn3	115.2 (2)
C114—C113—H113	120.1	O11—C5—O10	127.5 (4)
C112—C113—H113	120.1	O11—C5—C6	117.7 (3)
C115—C114—C113	120.8 (4)	O10—C5—C6	114.8 (3)
C115—C114—H114	119.6	O12—C6—O9	125.1 (4)
C113—C114—H114	119.6	O12—C6—C5	119.8 (3)
C114—C115—C116	119.2 (4)	O9—C6—C5	115.1 (3)
C114—C115—H115	120.4	C316—C311—C312	117.6 (4)
C116—C115—H115	120.4	C316—C311—Sn3	119.7 (3)
C115—C116—C111	121.4 (4)	C312—C311—Sn3	122.4 (3)
C115—C116—H116	119.3	C313—C312—C311	120.8 (4)
C111—C116—H116	119.3	C313—C312—H312	119.6
C122—C121—C126	118.3 (4)	C311—C312—H312	119.6
C122—C121—Sn1	122.8 (3)	C314—C313—C312	119.7 (4)
C126—C121—Sn1	118.8 (3)	C314—C313—H313	120.1
C121—C122—C123	120.5 (4)	C312—C313—H313	120.1
C121—C122—H122	119.7	C313—C314—C315	119.8 (4)
C123—C122—H122	119.7	C313—C314—H314	120.1
C124—C123—C122	120.6 (4)	C315—C314—H314	120.1
C124—C123—H123	119.7	C316—C315—C314	120.3 (4)
C122—C123—H123	119.7	C316—C315—H315	119.9
C123—C124—C125	119.8 (4)	C314—C315—H315	119.9
C123—C124—H124	120.1	C315—C316—C311	121.8 (4)
C125—C124—H124	120.1	C315—C316—H316	119.1
C124—C125—C126	119.6 (4)	C311—C316—H316	119.1
C124—C125—H125	120.2	C322—C321—C326	118.7 (4)
C126—C125—H125	120.2	C322—C321—Sn3	123.6 (3)
C125—C126—C121	121.2 (4)	C326—C321—Sn3	117.5 (3)
C125—C126—H126	119.4	C321—C322—C323	120.6 (4)
C121—C126—H126	119.4	C321—C322—H322	119.7
C136—C131—C132	117.5 (4)	C323—C322—H322	119.7
C136—C131—Sn1	122.2 (4)	C324—C323—C322	119.7 (4)
C132—C131—Sn1	120.1 (3)	C324—C323—H323	120.2
C133—C132—C131	122.5 (4)	C322—C323—H323	120.2
C133—C132—H132	118.7	C325—C324—C323	120.3 (4)
C131—C132—H132	118.7	C325—C324—H324	119.8
C132—C133—C134	118.9 (5)	C323—C324—H324	119.8
C132—C133—H133	120.6	C326—C325—C324	120.6 (4)
C134—C133—H133	120.6	C326—C325—H325	119.7
C135—C134—C133	119.5 (5)	C324—C325—H325	119.7
C135—C134—H134	120.2	C325—C326—C321	120.2 (4)
C133—C134—H134	120.2	C325—C326—H326	119.9
C134—C135—C136	121.0 (5)	C321—C326—H326	119.9
C134—C135—H135	119.5	C336—C331—C332	117.2 (3)
C136—C135—H135	119.5	C336—C331—Sn3	122.8 (3)
C135—C136—C131	120.5 (5)	C332—C331—Sn3	119.9 (3)
C135—C136—H136	119.7	C333—C332—C331	121.3 (4)
C131—C136—H136	119.7	C333—C332—H332	119.3
C141—N1—C151	114.4 (3)	C331—C332—H332	119.3
C141—N1—H1A	108.7	C334—C333—C332	119.7 (4)
C151—N1—H1A	108.7	C334—C333—H333	120.2
C141—N1—H1B	108.7	C332—C333—H333	120.2
C151—N1—H1B	108.7	C333—C334—C335	119.9 (4)
H1A—N1—H1B	107.6	C333—C334—H334	120.0
N1—C141—C142	110.7 (3)	C335—C334—H334	120.0
N1—C141—H14A	109.5	C336—C335—C334	119.8 (4)
C142—C141—H14A	109.5	C336—C335—H335	120.1
N1—C141—H14B	109.5	C334—C335—H335	120.1
C142—C141—H14B	109.5	C335—C336—C331	122.0 (4)
H14A—C141—H14B	108.1	C335—C336—H336	119.0
C143—C142—C147	118.7 (4)	C331—C336—H336	119.0
C143—C142—C141	121.2 (4)	C341—N3—C351	113.5 (3)
C147—C142—C141	120.1 (4)	C341—N3—H3A	108.9
C142—C143—C144	121.1 (4)	C351—N3—H3A	108.9
C142—C143—H143	119.4	C341—N3—H3B	108.9
C144—C143—H143	119.4	C351—N3—H3B	108.9
C145—C144—C143	119.4 (4)	H3A—N3—H3B	107.7
C145—C144—H144	120.3	N3—C341—C342	113.4 (3)
C143—C144—H144	120.3	N3—C341—H34A	108.9
C146—C145—C144	120.6 (4)	C342—C341—H34A	108.9
C146—C145—H145	119.7	N3—C341—H34B	108.9
C144—C145—H145	119.7	C342—C341—H34B	108.9
C145—C146—C147	120.7 (4)	H34A—C341—H34B	107.7
C145—C146—H146	119.6	C343—C342—C347	119.0 (4)
C147—C146—H146	119.6	C343—C342—C341	123.6 (4)
C146—C147—C142	119.5 (4)	C347—C342—C341	117.3 (4)
C146—C147—H147	120.3	C342—C343—C344	120.3 (4)
C142—C147—H147	120.3	C342—C343—H343	119.8
C152—C151—N1	111.1 (3)	C344—C343—H343	119.8
C152—C151—H15A	109.4	C345—C344—C343	120.4 (5)
N1—C151—H15A	109.4	C345—C344—H344	119.8
C152—C151—H15B	109.4	C343—C344—H344	119.8
N1—C151—H15B	109.4	C344—C345—C346	119.4 (5)
H15A—C151—H15B	108.0	C344—C345—H345	120.3
C153—C152—C157	118.5 (4)	C346—C345—H345	120.3
C153—C152—C151	121.7 (4)	C345—C346—C347	120.7 (5)
C157—C152—C151	119.8 (4)	C345—C346—H346	119.7
C154—C153—C152	120.9 (4)	C347—C346—H346	119.7
C154—C153—H153	119.5	C346—C347—C342	120.2 (5)
C152—C153—H153	119.5	C346—C347—H347	119.9
C153—C154—C155	119.7 (4)	C342—C347—H347	119.9
C153—C154—H154	120.2	N3—C351—C352	113.1 (3)
C155—C154—H154	120.2	N3—C351—H35A	109.0
C156—C155—C154	121.1 (5)	C352—C351—H35A	109.0
C156—C155—H155	119.4	N3—C351—H35B	109.0
C154—C155—H155	119.4	C352—C351—H35B	109.0
C155—C156—C157	118.5 (5)	H35A—C351—H35B	107.8
C155—C156—H156	120.8	C353—C352—C357	118.9 (4)
C157—C156—H156	120.8	C353—C352—C351	119.6 (4)
C152—C157—C156	121.3 (4)	C357—C352—C351	121.5 (4)
C152—C157—H157	119.4	C352—C353—C354	120.0 (5)
C156—C157—H157	119.4	C352—C353—H353	120.0
O6—Sn2—C221	121.57 (17)	C354—C353—H353	120.0
O6—Sn2—C211	116.99 (13)	C355—C354—C353	120.2 (5)
C221—Sn2—C211	115.19 (19)	C355—C354—H354	119.9
O6—Sn2—C231	84.98 (13)	C353—C354—H354	119.9
C221—Sn2—C231	107.13 (19)	C356—C355—C354	119.3 (5)
C211—Sn2—C231	103.03 (15)	C356—C355—H355	120.3
O6—Sn2—O5	72.71 (10)	C354—C355—H355	120.3
C221—Sn2—O5	86.49 (15)	C355—C356—C357	119.7 (5)
C211—Sn2—O5	86.21 (13)	C355—C356—H356	120.1
C231—Sn2—O5	157.64 (13)	C357—C356—H356	120.1
C3—O5—Sn2	115.9 (3)	C356—C357—C352	121.9 (5)
C4—O6—Sn2	120.8 (2)	C356—C357—H357	119.1
O7—C3—O5	127.2 (4)	C352—C357—H357	119.1
O7—C3—C4	117.8 (3)	O13—Sn4—C421	115.39 (14)
O5—C3—C4	114.9 (3)	O13—Sn4—C411	127.11 (15)
O8—C4—O6	124.0 (4)	C421—Sn4—C411	112.96 (15)
O8—C4—C3	120.5 (3)	O13—Sn4—C431	81.50 (13)
O6—C4—C3	115.6 (3)	C421—Sn4—C431	107.16 (15)
C212—C211—C216	118.2 (4)	C411—Sn4—C431	103.67 (16)
C212—C211—Sn2	124.0 (3)	O13—Sn4—O14	72.69 (11)
C216—C211—Sn2	117.8 (3)	C421—Sn4—O14	91.89 (14)
C211—C212—C213	119.9 (4)	C411—Sn4—O14	85.73 (14)
C211—C212—H212	120.1	C431—Sn4—O14	152.79 (13)
C213—C212—H212	120.1	C7—O13—Sn4	121.4 (3)
C214—C213—C212	121.3 (4)	C8—O14—Sn4	116.1 (3)
C214—C213—H213	119.4	O15—C7—O13	125.5 (4)
C212—C213—H213	119.4	O15—C7—C8	120.2 (4)
C213—C214—C215	119.5 (4)	O13—C7—C8	114.3 (4)
C213—C214—H214	120.2	O16—C8—O14	127.6 (4)
C215—C214—H214	120.2	O16—C8—C7	117.3 (4)
C216—C215—C214	119.8 (4)	O14—C8—C7	115.1 (4)
C216—C215—H215	120.1	C416—C411—C412	118.6 (4)
C214—C215—H215	120.1	C416—C411—Sn4	115.9 (3)
C215—C216—C211	121.3 (4)	C412—C411—Sn4	125.4 (3)
C215—C216—H216	119.3	C413—C412—C411	119.4 (4)
C211—C216—H216	119.3	C413—C412—H412	120.3
C22A—C221—C226	94.4 (8)	C411—C412—H412	120.3
C22A—C221—C222	50.2 (7)	C414—C413—C412	122.0 (5)
C226—C221—C222	116.2 (5)	C414—C413—H413	119.0
C22A—C221—C26A	115.5 (9)	C412—C413—H413	119.0
C226—C221—C26A	36.2 (7)	C413—C414—C415	119.4 (5)
C222—C221—C26A	106.2 (7)	C413—C414—H414	120.3
C22A—C221—Sn2	117.7 (7)	C415—C414—H414	120.3
C226—C221—Sn2	118.9 (4)	C416—C415—C414	119.1 (4)
C222—C221—Sn2	124.4 (4)	C416—C415—H415	120.5
C26A—C221—Sn2	122.2 (6)	C414—C415—H415	120.5
C223—C222—C221	121.9 (6)	C411—C416—C415	121.5 (4)
C223—C222—H222	119.0	C411—C416—H416	119.3
C221—C222—H222	119.0	C415—C416—H416	119.3
C222—C223—C224	117.8 (6)	C426—C421—C422	118.5 (4)
C222—C223—H223	121.1	C426—C421—Sn4	118.8 (3)
C224—C223—H223	121.1	C422—C421—Sn4	121.9 (3)
C225—C224—C25A	30.9 (8)	C423—C422—C421	120.1 (4)
C225—C224—C23A	94.9 (7)	C423—C422—H422	120.0
C25A—C224—C23A	115.4 (9)	C421—C422—H422	120.0
C225—C224—C223	120.3 (6)	C422—C423—C424	120.2 (4)
C25A—C224—C223	113.5 (8)	C422—C423—H423	119.9
C23A—C224—C223	52.2 (7)	C424—C423—H423	119.9
C225—C224—H224	119.8	C425—C424—C423	120.3 (4)
C25A—C224—H224	117.5	C425—C424—H424	119.9
C23A—C224—H224	122.0	C423—C424—H424	119.9
C223—C224—H224	119.8	C424—C425—C426	119.8 (4)
C224—C225—C226	121.5 (6)	C424—C425—H425	120.1
C224—C225—H225	119.2	C426—C425—H425	120.1
C226—C225—H225	119.2	C421—C426—C425	121.1 (4)
C221—C226—C225	122.1 (6)	C421—C426—H426	119.5
C221—C226—H226	119.0	C425—C426—H426	119.5
C225—C226—H226	119.0	C436—C431—C432	117.6 (4)
C221—C22A—C23A	121.6 (12)	C436—C431—Sn4	119.8 (3)
C221—C22A—H22A	119.2	C432—C431—Sn4	122.1 (3)
C23A—C22A—H22A	119.2	C433—C432—C431	121.4 (4)
C22A—C23A—C224	119.9 (12)	C433—C432—H432	119.3
C22A—C23A—H23A	120.1	C431—C432—H432	119.3
C224—C23A—H23A	120.1	C432—C433—C434	119.3 (4)
C224—C25A—C26A	117.8 (11)	C432—C433—H433	120.4
C224—C25A—H25A	121.1	C434—C433—H433	120.4
C26A—C25A—H25A	121.1	C435—C434—C433	120.2 (4)
C25A—C26A—C221	121.8 (12)	C435—C434—H434	119.9
C25A—C26A—H26A	119.1	C433—C434—H434	119.9
C221—C26A—H26A	119.1	C434—C435—C436	119.9 (4)
C231—C232—C233	120.8 (5)	C434—C435—H435	120.1
C231—C232—H232	119.6	C436—C435—H435	120.1
C233—C232—H232	119.6	C431—C436—C435	121.6 (4)
C234—C233—C232	121.3 (5)	C431—C436—H436	119.2
C234—C233—H233	119.4	C435—C436—H436	119.2
C232—C233—H233	119.4	C451—N4—C441	114.6 (3)
C232—C231—C236	115.9 (4)	C451—N4—H4A	108.6
C232—C231—Sn2	124.8 (3)	C441—N4—H4A	108.6
C236—C231—Sn2	119.3 (3)	C451—N4—H4B	108.6
C233—C234—C235	119.3 (5)	C441—N4—H4B	108.6
C233—C234—H234	120.3	H4A—N4—H4B	107.6
C235—C234—H234	120.3	C442—C441—N4	112.8 (3)
C234—C235—C236	120.0 (5)	C442—C441—H44A	109.0
C234—C235—H235	120.0	N4—C441—H44A	109.0
C236—C235—H235	120.0	C442—C441—H44B	109.0
C235—C236—C231	122.6 (4)	N4—C441—H44B	109.0
C235—C236—H236	118.7	H44A—C441—H44B	107.8
C231—C236—H236	118.7	C443—C442—C447	118.7 (4)
C241—N2—C251	114.7 (3)	C443—C442—C441	121.9 (4)
C241—N2—H2A	108.6	C447—C442—C441	119.4 (4)
C251—N2—H2A	108.6	C442—C443—C444	120.8 (4)
C241—N2—H2B	108.6	C442—C443—H443	119.6
C251—N2—H2B	108.6	C444—C443—H443	119.6
H2A—N2—H2B	107.6	C445—C444—C443	119.7 (5)
N2—C241—C242	113.0 (3)	C445—C444—H444	120.2
N2—C241—H24A	109.0	C443—C444—H444	120.2
C242—C241—H24A	109.0	C444—C445—C446	120.5 (5)
N2—C241—H24B	109.0	C444—C445—H445	119.7
C242—C241—H24B	109.0	C446—C445—H445	119.7
H24A—C241—H24B	107.8	C447—C446—C445	119.4 (5)
C247—C242—C243	119.2 (4)	C447—C446—H446	120.3
C247—C242—C241	118.1 (4)	C445—C446—H446	120.3
C243—C242—C241	122.6 (3)	C446—C447—C442	120.8 (5)
C244—C243—C242	120.5 (4)	C446—C447—H447	119.6
C244—C243—H243	119.8	C442—C447—H447	119.6
C242—C243—H243	119.8	N4—C451—C452	112.9 (3)
C243—C244—C245	120.4 (4)	N4—C451—H45A	109.0
C243—C244—H244	119.8	C452—C451—H45A	109.0
C245—C244—H244	119.8	N4—C451—H45B	109.0
C246—C245—C244	119.0 (4)	C452—C451—H45B	109.0
C246—C245—H245	120.5	H45A—C451—H45B	107.8
C244—C245—H245	120.5	C453—C452—C457	118.9 (4)
C245—C246—C247	120.6 (4)	C453—C452—C451	122.3 (4)
C245—C246—H246	119.7	C457—C452—C451	118.5 (4)
C247—C246—H246	119.7	C452—C453—C454	120.5 (4)
C242—C247—C246	120.2 (4)	C452—C453—H453	119.7
C242—C247—H247	119.9	C454—C453—H453	119.7
C246—C247—H247	119.9	C455—C454—C453	119.9 (4)
C252—C251—N2	113.1 (3)	C455—C454—H454	120.0
C252—C251—H25B	108.9	C453—C454—H454	120.0
N2—C251—H25B	108.9	C454—C455—C456	119.5 (4)
C252—C251—H25C	108.9	C454—C455—H455	120.3
N2—C251—H25C	108.9	C456—C455—H455	120.3
H25B—C251—H25C	107.8	C455—C456—C457	120.8 (4)
C257—C252—C253	118.6 (4)	C455—C456—H456	119.6
C257—C252—C251	119.8 (4)	C457—C456—H456	119.6
C253—C252—C251	121.5 (4)	C456—C457—C452	120.3 (4)
C254—C253—C252	120.7 (4)	C456—C457—H457	119.8
C254—C253—H253	119.7	C452—C457—H457	119.8

### Hydrogen-bond geometry (Å, º)

**Table:**

*D*—H···*A*	*D*—H	H···*A*	*D*···*A*	*D*—H···*A*
N1—H1*A*···O4	0.92	1.94	2.847 (4)	171
N1—H1*B*···O15	0.92	2.26	2.902 (4)	126
N1—H1*B*···O16	0.92	1.98	2.805 (4)	148
N2—H2*A*···O7	0.92	1.92	2.795 (4)	159
N2—H2*B*···O11	0.92	2.05	2.857 (4)	146
N2—H2*B*···O12	0.92	2.27	2.925 (4)	128
N3—H3*A*···O7	0.92	2.21	2.951 (4)	137
N3—H3*A*···O8	0.92	2.08	2.813 (4)	136
N3—H3*B*···O11	0.92	1.91	2.816 (4)	169
N4—H4*A*···O3	0.92	2.15	2.843 (4)	131
N4—H4*A*···O4	0.92	2.10	2.843 (4)	137
N4—H4*B*···O16	0.92	1.84	2.755 (4)	177
